# Current Challenges and Limitations in Antibody-Based Detection of Citrullinated Histones

**DOI:** 10.3389/fimmu.2016.00528

**Published:** 2016-11-25

**Authors:** Indira Neeli, Marko Radic

**Affiliations:** ^1^Department of Microbiology, Immunology and Biochemistry, University of Tennessee Health Science Center, Memphis, TN, USA

**Keywords:** NETs, NETosis, deimination, peptidylarginine deiminase, antibodies, immunodetection, citrulline

## Abstract

Studies on NETosis demand reliable and convenient markers to monitor the progress of this form of cell death. Because a determining step in the release of nuclear chromatin NETs requires the conversion of arginine residues to citrulline residues in histones by peptidylarginine deiminase, citrullinated histones can provide such a marker. Here, we evaluate antibody reagents for the detection of citrulline residues in histones and observe alarming differences between commercial antisera and mouse and rabbit monoclonal antibodies in their ability to detect their nominal target residues. Differences between antibodies that are currently used to detect citrulline residues in histones could jeopardize efforts to reach a scientific consensus and instead lead to inconsistent and even conflicting conclusions regarding the regulation of histone deimination. Our results will assist others in planning their initial or ongoing studies on peptidylarginine deiminase activity with the use of currently available antibodies. Furthermore, we argue that, along with the careful attention to experimental conditions and calcium concentrations, validated antibody reagents are urgently needed to avoid possible setbacks in the research on NETosis.

The years since 2004 have seen an explosive rise in interest in neutrophil cell death mechanism. Much of the interest was sparked by the discovery of NETosis by Brinkmann et al. (1), which implicated this unique form of cell death in infectious and autoimmune diseases. It is difficult to adequately summarize all the remarkable discoveries since that landmark paper was published. Some examples include the relation between NETosis, autophagy (2), apoptosis (3), necroptosis (4), and granzyme-mediated cell death (5). Insights into the regulation of NETosis have defined the roles of cell surface receptors (6), protein kinases (7), elastase, and myeloperoxidase (8). The participation of NETs has been demonstrated in inflammatory diseases such as acute lung injury (9), thrombosis (10), cystic fibrosis (11), vasculitis (12), gout (13), diabetes (14), and even Alzheimer’s (15). NETosis also directly contributes to the induction of autoantibodies in major autoimmune diseases such as rheumatoid arthritis (16) and systemic lupus erythematosus (17). The process of chromatin NET release may not be unique to vertebrates, as plants (18) and slime molds (19) have mechanisms to release nuclear chromatin under specific circumstances. Subtypes of NETs have been reported and an important form of NETosis has been identified in which neutrophils release nuclear DNA but continue certain functions such as chemotaxis despite the casting of NETs (20). Similarly, NETs consisting of mitochondrial DNA have been characterized that may be compatible with continued functions of neutrophils (21–23). NETs are also released by other granulocytes (24), macrophages (25), and mast cells (26).

NETosis is characterized by large-scale morphological transitions that can be seen as the swelling of the lobed nucleus, the rupture of the nuclear envelope, and the release of NETs that can stretch to many times the size of a single neutrophil (27). Thus, NETosis has been measured by automated image analysis (28), by quantitative fluorescence activated cytometry (29), and by immunofluorescence detection of colocalized DNA and neutrophil granule components (30). The detection of deiminated histones represents an important hallmark of NETosis because the enzyme responsible for histone modification, peptidylarginine deiminase IV (PAD4), is activated on a massive scale during the progress of NETosis (31). Indeed, the activation of PAD4 is intimately linked with the production of autoantibodies to citrullinated proteins. These antibodies are sensitive and predictive criteria in a number of autoimmune diseases (3, 32–34). Importantly, several groups of researchers have provided evidence that NET release is dramatically impaired by the genetic deficiency (35) or the pharmacological inhibition of PAD4 (36), and that PAD4 inhibitors offer promising starting points to develop autoimmune disease therapies (37, 38).

The detection of deiminated histones *in vivo* has been interpreted as evidence for NETs, as may occur in nephritis associated with vasculitis (39), thrombus formation (29), lung injury (40), and due to alum adjuvant stimulation (41). Detection of histone deimination has also been helpful in testing aspects of PAD4 regulation (42). However, inconsistencies between results reported by different labs have also appeared in the literature. For example, one widely used stimulus, PMA, has resulted in conflicting results in the literature. Thus, PMA was observed to induce deimination (35) or suppress deimination (7). Our result was surprising due to the frequent use of PMA to induce the release of NETs, and the common assumption that PAD activity is required for NET release to occur. Therefore, we carefully analyzed the phenomenon and observed that PMA also suppressed histone deimination in the presence of A23187 ionophore, a compound that by itself is a strong inducer of deimination (7). We further established that PMA inhibited PAD4 *via* activation of PKCα/β. Our results have been confirmed by Douda et al. who characterized two alternate forms of neutrophil cell death leading to NET release (42). In addition, apoptosis induction may block histone deimination (3) or promote it (5). Certainly, the conflicting results could be explained by various differences in the execution of these experiments, including details of buffers and media used during stimulation, yet one testable possibility was that the reagents for detecting deimination were inconsistent.

The most convenient way to measure histone deimination is with antibodies that recognize citrulline residues within their specific antigenic epitope. Various commercial antibodies based on polyclonal sera or monoclonal antibodies (Mab) are available for immunochemical detection of deiminated histones. Caution is advised, as polyclonal antisera may differ from animal to animal according to stochastic events that generate antibody specificity. Conversely, Mabs can be highly specific but may also be sensitive to subtle changes in the epitope due to contributions from flanking residues.

Thus, we set out to assess the reliability and consistency of different commercial antibodies against deiminated histones. To provide samples for our analysis, we prepared whole cell lysates from freshly isolated human neutrophils that were treated with diverse stimuli to induce or suppress histone deimination. For a commonly accepted baseline, we analyzed the lysates with antibodies to diacetyl monoxime/antipyrine-modified citrullines (Figure 1A), using a detection kit from Millipore (43). To assess the quantity and integrity of the core histones, we used antibodies to total histone H3 (Cell Signaling Technologies, #4620S) to generate the blot shown in Figure 1B. All incubations, except for unstimulated neutrophils (lanes 1), contained 200 μM calcium in addition to the diverse stimuli. In all cases, the yields of intact H3 were comparable, except in samples treated with 20 μM chelerythrine along with ionophore (lanes 5) or lanthanum 200 μM (lanes 11), which showed partial cleavage of H3 (Figure 1B). Most treatments induced moderate to high levels of histone deimination, except, as previously reported, 20 μM chelerythrine and 20 nM PMA (lanes 5 and 9, Figure 1A), which showed little to no deimination (7). The most intense deimination was observed in cells that were incubated with 5 μM chelerythrine and ionophore (lanes 4), and cells incubated with lanthanum (Figure 1A).

**Figure 1:**
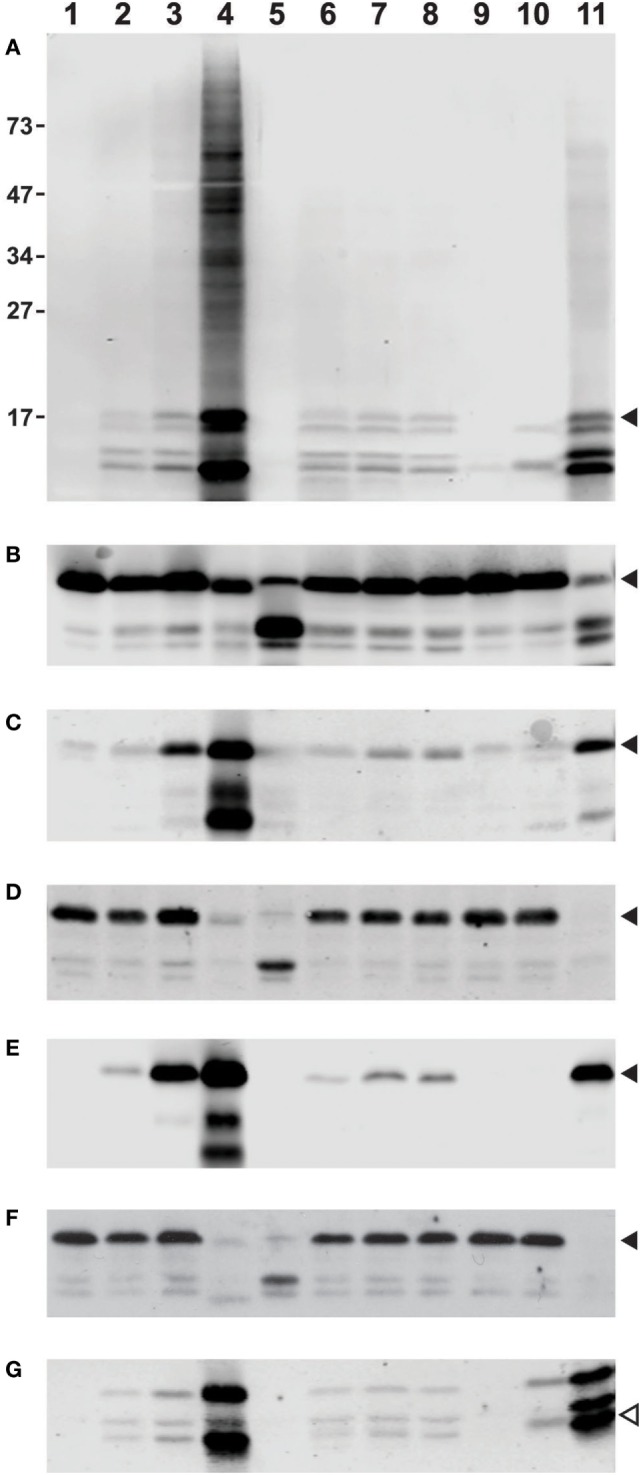
**Differences among commercial antibody-based reagents for the detection of deiminated core histones**. Human neutrophils were purified from healthy donor blood according to published procedures and incubated for 2 h in HBSS (lane 1) or HBSS with 200 μM calcium chloride (lane 2) and A27632 ionophore (lane 3), ionophore with 5 μM chelerythrine (lane 4), or 20 μM chelerythrine (lane 5). Cells were also incubated in the presence of 200 μM calcium chloride and hydroxyapatite (lane 6) with added LPS (lane 7), or fMLP (lane 8), 20 nM PMA (lane 9), manganese chloride (lane 10), or lanthanum chloride (lane 11). The whole cell lysates were run on a denaturing 12% PAGE and blotted to nitrocellulose membrane prior to reaction with diacetyl monoxime/antipyrine and detection of modified citrullines with the antibody and following instructions from Millipore **(A)**. Alternatively, blots were blocked for 1 h at RT with 5% bovine serum albumin (BSA) or 5% milk in TBST [Tris-buffered saline (TBS) and Tween 20, 25 mM Tris (pH 7.2), 150 mM NaCl, and 0.1% Tween 20] and rinsed before overnight incubation at 4°C with a dilution of primary Abs (as recommended by the supplier) in 2.5% BSA in TBST. Subsequently, membranes were washed and incubated for 1 h with donkey anti-rabbit secondary Ab IR800 (catalog #925-32213) or goat anti-mouse (catalog #926-32210) as secondary antibodies available from LI-COR, washed three times with TBST and twice with TBS alone and developed on an Odyssey imaging system. Blots were reacted with antibodies to total histone H3, obtained from Cell Signaling Technologies **(B)**, rabbit antisera to citrullines at positions 2, 8, and 17 of histone H3, Abcam, catalog #ab5103 **(C)**, mouse Mab to citrullines at the same positions, also from Abcam, clone 7C10 **(D)**, a rabbit Mab to citrulline at position 2 of H3, Abcam, catalog #176843 **(E)**, and a mouse IgM Mab to poly-citrulline, F95 from Millipore **(F)**. For comparison, we used a rabbit antiserum to citrullines in the amino terminus of H4, supplied by Millipore under #07-596 **(G)**. Filled arrowheads indicate position of H3 on the membrane, whereas the open symbol points to the position of H4. The membrane in **(A)** displays additional reactivity to proteins of slower mobility on the gel (lane 4). The distance to which marker proteins had migrated and their masses in kilodaltons are indicated on the margin of **(A)**.

A widely used polyclonal antibody that recognizes histone H3 with citrulline residues at positions 2, 8, and 17 (Abcam #ab5103, Lot GR247556) could detect histone deimination (Figure 1C) in roughly similar measure as the modified citrulline antibody (Figure 1A), although the Abcam antibody also reacted weakly with unstimulated and 20 nM PMA-treated neutrophil lysates. We have compared different lots of this antibody and observed lot-to-lot variability (data not shown). This lot detected a small increase in deimination in the presence of extracellular calcium (lane 2), presumably reflecting the enzyme’s requirement for calcium. However, a significant increase was noted, once ionophore opened access for calcium across the plasma membrane (lane 3). There was an even greater enhancement of deimination with 5 μM chelerythrine (lane 4), a selective inhibitor of certain protein kinase C isozymes. Raising the concentration of chelerythrine to 20 μM impaired histone deimination (lane 5). Mechanical damage to the plasma membrane, as may be induced by frustrated phagocytosis of hydroxyapatite crystals, also enhanced deimination (lane 6, Figure 1C), and this effect could be further stimulated by addition of LPS or fMLP (lanes 7 and 8). In contrast, incubation in calcium with 20 nM PMA did not stimulate deimination (lane 9), despite the fact that NETosis is greatly induced by this compound (data not shown). Manganese, and, more intensely, the combination of calcium and lanthanum (lanes 10 and 11), induced deimination.

To compare the rabbit antisera to MAbs, which represent more stable immunological reagents, we examined three commercially available MAbs that were promoted as detection reagents for citrulline residues. A mouse MAb to citrullinated histone H3 (Abcam, #ab80256, clone 7C10), which was listed as reacting against H3 with citrullines at positions 2, 8, and 17, showed a drastically different pattern of reactivity than the antisera used above (Figure 1D). In this instance, the antibody appeared insensitive to large increases in deimination, as the antibody did not distinguish between unstimulated lysates and lysates prepared following the incubation with ionophore. In fact, the signal was low to absent in the samples treated with 5 μM chelerythrine or lanthanum, which gave the most intense signals with the two antisera used above (Figures 1A,C).

In contrast, a rabbit Mab from the same supplier (Abcam, #176843) that binds to citrulline at the second position of histone H3, could detect a strong increase of H3 deimination with ionophore treatment and a further enhancement with 5 μM chelerythrine (Figure 1E). This antibody also gave a strong signal with the lysate treated with lanthanum, although it showed only a marginal signal increase with lysates from cells incubated with hydroxyapatite in combination with LPS or fMLP, and no reaction with manganese or PMA-treated cell lysates.

Results with F95 (Figure 1F), a mouse IgM reported to recognize citrullines in a context-independent manner (Millipore, MABN328), further emphasized the drastic differences between MAbs to citrulline epitopes. The same neutrophil lysates as used in the examples above showed no increase in histone deimination with addition of ionophore or hydroxyapatite treatment in calcium buffer. Thus, the signal that was detected with histones from unstimulated cells showed little to no modulation following induction of deimination. In addition, enhanced deimination that was induced with 5 μM chelerythrine or with lanthanum was nearly completely undetectable (Figure 1F). These results were inconsistent with the previous observations. On a longer exposure, F95 IgM could detect citrullination of other proteins, as seen by the increased reactivity with proteins of increased molecular weights (data not shown). Thus, there is a specific problem with the detection of citrullines in histones by F95.

To extend our analysis to antibodies to other deiminated core histones, we tested a rabbit antiserum to the citrullinated amino terminus of histone H4 (Millipore, #07-596). This polyclonal antibody detected the enhanced level of deimination in the presence of 5 μM chelerythrine and lanthanum (Figure 1G), but it was somewhat less sensitive to calcium/ionophore or hydroxyapatite than the mouse antiserum to H3 (Figure 1C). This antibody showed cross-reactivity with proteins of greater molecular weight than H4, as acknowledged by the supplier, which could be due to a shared amino acid motif between histone H4 and H2A. Nevertheless, the results with this antibody were comparable to those obtained with the tri-citrullinated H3 antibody (Figure 1C) and the rabbit MAb (Figure 1E).

In conclusion, we uncover a surprising level of inconsistency between commercially available antibodies to citrulline-containing epitopes. Others have recently pointed out the need for commercial suppliers of antibodies to more carefully assay and validate different lots of antibodies (44–48). This is especially relevant for antibodies to histone post-translational modifications, as histones incur numerous modifications that are relevant for the functional properties of chromatin. An International Working Group for Antibody Validation was recently convened and published a set of recommendations for antibody validation because of the enormous losses of research funds due to the inconsistent data that arise based on currently available antibody reagents (49). One useful resource is an online repository of antibody-binding specificities that currently lists over 100 antibodies to histone post-translational modifications (47). Our results argue for a cautious approach to interpretations of any single antibody for the determination of deiminase activity in neutrophils or other cell types of interest. Although the modified citrulline antibody is more complicated to use than the other antibodies, a prudent approach may include the use of this antibody for comparison to the other reagents. Other ways of detecting citrulline have been reported, but they may require more sophisticated equipment or more complicated analyses. Generally, the current challenge in the field of NETosis research with regard to histone deimination is acute and requires reliable, accepted, and broadly available reagents. Increased efforts to isolate monoclonal mouse or rabbit Ab, including possibly by recombinant methods, should be promoted. Notably, advances in phage display technology have led to the discovery of antibodies that are specific for various post-translational protein modifications, including acetylation, phosphorylation, methylation, and citrullination (50) and that allow their efficient conversion into IgG molecules (51).

## Author Contributions

IN conducted the laboratory research and assisted in the drafting and revision of the manuscript. MR conceived the experimental approach, interpreted the data, and wrote the manuscript.

## Conflict of Interest Statement

The authors declare that the research was conducted in the absence of any commercial or financial relationships that could be construed as a potential conflict of interest.
